# Exploring the Domain of Emotional Intelligence in Organizations: Bibliometrics, Content Analyses, Framework Development, and Research Agenda

**DOI:** 10.3389/fpsyg.2022.810507

**Published:** 2022-03-07

**Authors:** Baobao Dong, Xing Peng, Na Jiang

**Affiliations:** ^1^School of Business and Management, Jilin University, Changchun, China; ^2^School of Music, Dance and Drama, Changchun Humanities and Sciences College, Changchun, China

**Keywords:** emotional intelligence, group emotional intelligence, framework development, bibliometric, research agenda

## Abstract

Emotion is a kind of micro foundation that can affect human behaviors even in the digital era. Emotional intelligence (EI) is an important psychological factor that affects the growth and development of organizations from the view of emotion. Based on current bodies of literature, a comprehensive review of EI can contribute to its theory development in organization research and facilitate EI research burgeoning. We visualize the landscape of EI by analyzing 1,996 articles with CiteSpace their concepts, dimensions, and measurement. We propose two specific mechanisms, which clarify how individuals with high EI use emotional information to influence themselves and others. Following this, we develop a theoretical framework of EI at levels of individual, team, and organization. Finally, future directions and research agenda are addressed. This research contributes to the literature of EI and provides practical insight for practitioners.

## Introduction

Emotion is fundamental to human experiences influencing our daily activities such as cognition, communication, learning, and decision making. For centuries, psychologists have tried to understand and define emotions. Recently, emotional intelligence (herein referred to as EI), as a special unique resource within organizations, has gained attention from scholars and practitioners. Recent studies highlight the importance of EI as a predictor in important domains, such as psychology (e.g., job satisfaction, self-efficacy), behaviors (e.g., organizational citizenship behavior, workplace deviant behavior, ethical behavior) and work outcomes (e.g., job performance, leadership effectiveness, career success) (Wong and Law, 2002; Brackett et al., 2004; Landy, 2005; Momm et al., 2014; Tuncdogan et al., 2017). As such, we argue that EI is an important factor affecting organizational development and future growth.

Emotional intelligence, as an individual-level variable, means affective tendency to effectively use emotional information to achieve expected results (Bell, 2007). Members in an organization with high EI can successfully affect the social environment at work and achieve high performance by regulating their emotions (Momm et al., 2014), which is also considered as the main reason why early studies on EI focused on the individual level. However, it should be noted that an organization is a social structure interwoven with relationships, and the flow of emotional information will not only affect individual behavior but also have cross-level effects. On the one hand, because decisions and behaviors of organizational members are always affected by emotional factors, EI of high-power members may have a significant impact on team or organizational effectiveness (Azouzi and Jarboui, 2014). On the other hand, in social communication, EI may affect emotional or behavioral responses of others. Individual EI can also be aggregated into a higher level of group EI. Such group norms that deal with emotions effectively and flexibly not only regulate the emotional state of individual members or teams inward but also affect the atmosphere of other teams or organizations outward, thus producing cross-level effects (Druskat and Wolff, 2001). As an antecedent of organizational performance, EI may be regarded as a hidden “driving force” affecting organizational growth (Momm et al., 2014).

However, since the concept of EI was proposed in the 1990s, more and more scholars began to pay attention to EI, but it cannot be ignored that the literature is still limited in the following two ways. First, the concept and measurement of EI are still controversial. There are various concepts of EI under different theoretical frameworks, and its measurements are not completely consistent, which leads to inconsistent results (Landy, 2005; Tuncdogan et al., 2017; MacCann et al., 2020). Second, the scope of research topics is a little narrow and concentrated. EI research mostly focuses on the workplace, and its influence at the level of team and organization is relatively single. As an individual-level variable in an organization, the influence of EI on all levels of an organization may be more complex, and future studies need to further explore the differentiated process and influences of EI at different levels.

Therefore, we believe that a review of EI should be conducted to understand the current research situation and trend of EI. First, we present the development status of EI in the field of organization by analyzing bodies of literature. We use big data analysis methods, such as CiteSpace, to conduct quantitative analysis and present the landscape and evolution of EI. Second, we review the definitions, dimensions, and measurements of EI. Third, we establish and analyze two emotional influence mechanisms of individuals with high EI, which illustrate how individuals with high EI use emotional information to influence themselves and others. Fourth, after reviewing the existing bodies of literature on EI, we construct a theoretical framework focusing on the impact of EI and its corresponding moderating effect. Finally, we propose research agenda for future research.

## Visualizing the Landscape of Emotional Intelligence

Based on bibliometrics, CiteSpace is used to analyze the trend of EI research. The principle of collecting data is as follows. First, data are collected from the Web of Science database ranging from 1990 to 2020. The year 1990 is the base year, because the concept of EI was formally introduced by Salovey and Mayer in 1990. Second, we choose bodies of literature using terms such as “emotional intelligence” and “emotional ability” in the subject bar, set the operator to “OR,” and retain empirical articles and reviews. In addition, by screening literature topics, we select relevant literature in the fields of economics, management, and sociology (e.g., “psychology social,” “management,” “psychology applied,” “behavioral sciences,” “business,” “communication,” and “economics”). Finally, a total of 1,996 articles are retrieved as research objects by manual inspection and exclusion of irrelevant ones.

In order to clearly and intuitively display the full picture of EI, we conducted an analysis on views of publication, journals, scholars, node literature, and cooperation network.

### Analysis of Publications

**FIGURE 1:**
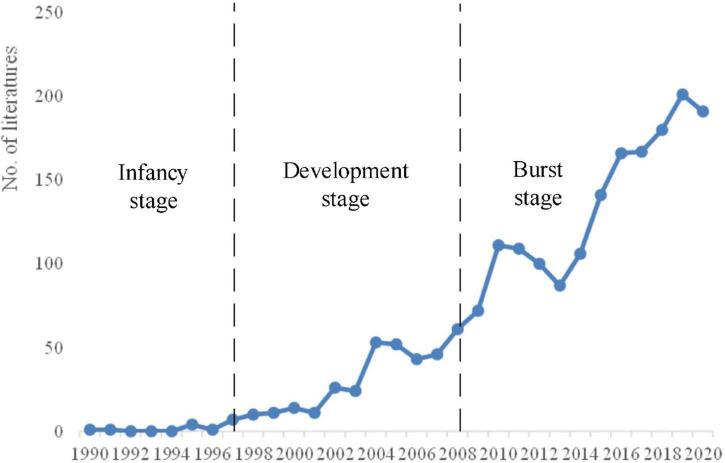
Chart of output trends in emotional intelligence (EI).

Review on EI is of help to provide a visualized picture of the popularity and trend of EI research. Figure 1 shows a total of 1,996 records that were published in the past 30 years *via* Web of Science. There is an increasing trend in publication, and it can be divided into three stages: infancy stage (1990–1997), development stage (1997–2008), and burst stage (2008–2020). During the infancy stage, it was controversial whether EI coincided with the concept of personal characteristics or cognitive intelligence in a wide range. Some scholars were skeptical about the research value of EI. Therefore, there were relatively few studies on EI in this stage. The field did not experience much growth until 1997. Scholars have started to pay their attention to EI since the ability model and mixed model of EI were first proposed in1997. The increasing bodies of literature provided evidence that EI had attracted an extraordinary attention from numerous scholars who focused on the concept and dimensions of EI and began to explore its effects. During its burst stage, the maturity of measurement was conducive to empirical research in this field, and publication showed a “blowout” growth.

### Analysis of Journals and Scholars

In terms of citation or centrality, Table 1 shows the top ten journals that publish EI research, which reflects that these journals show more in-depth research. As indicated in Table 1, the top ten journals include Journal of Organizational Behavior, Academy of Management Journal and other journals belonging to UTD24 and FT50, which shows that international journals are interested in EI. Table 2 shows the top ten scholars based on citation or centrality, which can help scholars understand the development and direction of EI.

**TABLE 1:** Ranking of journals based on citation rate/centrality (top 10).

Ranking	Journal	Citation rate	Journal	Centrality
1	Personality and Individual Differences	942	Emotion	0.17
2	The Journal of Applied Psychology	583	Personality and Social Psychology Bulletin	0.13
3	Journal of Organizational Behavior	439	Journal of Personality Assessment	0.13
4	Journal of Personality and Social Psychology	399	Personality and Individual Differences	0.11
5	Emotion	378	The Journal of Applied Psychology	0.11
6	Leadership Quarterly	292	Journal of Organizational Behavior	0.10
7	Academy of Management Journal	266	Cognition and Emotion	0.10
8	Annual Review of Psychology	256	Psychological Bulletin	0.10
9	Journal of Vocational Behavior	242	Journal of Management	0.09
10	Cognition and Emotion	227	Leadership and Organization Development Journal	0.09

**TABLE 2:** Ranking of authors based on citation rate/centrality (top 10).

Ranking	Authors	Citation rate	Authors	Centrality
1	Mayer, John D.	407	Petrides, K. V.	0.21
2	Petrides, K. V.	337	Mayer, John D.	0.14
3	Schutte, Nicola S.	196	Cote, Stephane	0.14
4	Brackett, Marc A.	182	Salovey, Peter	0.13
5	Joseph, Dana L.	181	Schutte, Nicola S.	0.12
6	Zeidner, M.	172	Elfenbein, Hillary Anger	0.10
7	Goleman, Daniel	161	Brackett, Marc A.	0.09
8	Cote, Stephane	139	Austin, Elizabeth J.	0.08
9	Austin, Elizabeth J.	136	Ashkanasy, Neal M.	0.08
10	Bar-On, Roi	132	Judge, Timothy A.	0.07

### Analysis of Key Node Literature

Table 3 shows the top ten cited key node bodies of literature in EI field that explain the core constructs of EI and mainly cover conceptual dimensions, relationship among dimensions, and measurement scales. For instance, Mayer et al. (1999) and Petrides et al. (2007) provide insight into the concept and nature of EI. Joseph and Newman (2010) explored relationships among the dimensions of EI and proposed a cascading model. Schutte et al. (1998), Brackett and Mayer (2003), Law et al. (2004), and Van Rooy and Viswesvaran (2004) conducted tests on measurement scales and content validity of EI. These node bodies of literature are conducive to subsequent theoretical development and empirical studies.

**TABLE 3:** Studies on emotional intelligence (EI) based on co-citation analysis from 1990 to 2020 (top 10).

References	Title	Journal
Joseph and Newman, 2010	Emotional intelligence: An integrative meta-analysis and cascading model	Journal of Applied Psychology
Mayer et al., 2008	Human abilities: Emotional intelligence	Annual Review of Psychology
O’Boyle et al., 2011	The relation between emotional intelligence and job performance: A meta-analysis	Journal of Organizational Behavior
Petrides et al., 2007	The location of trait emotional intelligence in personality factor space	British Journal of Psychology
Mayer et al., 1999	Emotional intelligence meets traditional standards for an intelligence	Intelligence
Brackett and Mayer, 2003	Convergent, discriminant, and incremental validity of competing measures of emotional intelligence	Personality and Social Psychology Bulletin
Martins et al., 2010	A comprehensive meta-analysis of the relationship between emotional intelligence and health	Personality and Individual Differences
Schutte et al., 1998	Development and validation of a measure of emotional intelligence	Personality and Individual Differences
Van Rooy and Viswesvaran, 2004	Emotional intelligence: A meta-analytic investigation of predictive validity and nomological net	Journal of Vocational Behavior
Law et al., 2004	The construct and criterion validity of emotional intelligence and its potential utility for management studies	Journal of Applied Psychology

*Source: Collated according to relevant literature.*

### Analysis of Cooperation Network

**FIGURE 2:**
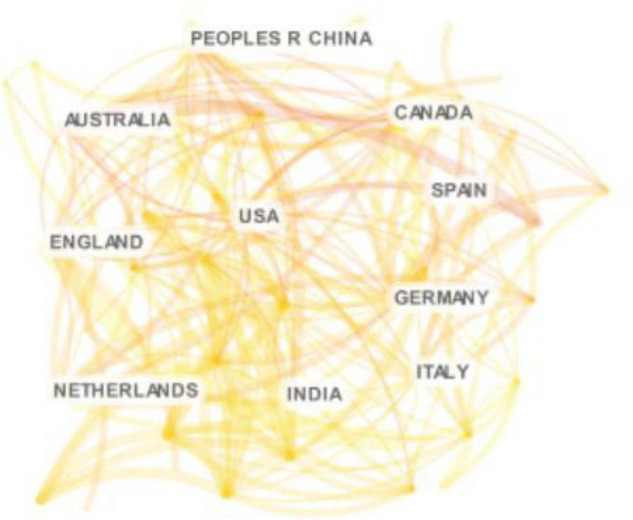
Countries/regions of cooperative networks of emotional intelligence research in organization field from 1990 to 2020.

Figure 2 shows a distribution of a country-based cooperation network. The top five countries ranked by output of EI literature are the United States (690), the United Kingdom (212), Australia (195), China (136), and Canada (130). In terms of publications, China has gradually begun to pay attention to EI, but the number is still quite different from that of the United States, the United Kingdom and other countries that lead the frontier of research on EI. In terms of centrality, China ranked 14th with 0.06, much lower than that of the United Kingdom (0.26) and the United States (0.21) which indicates that important future endeavors to address significant gaps in EI research between China and other countries is necessary.

## Definition, Dimension, and Measurement of Emotional Intelligence

### Definition of Emotional Intelligence

Compared with intelligence or personality, EI is a relatively new construct. Based on social intelligence theory (Thorndike, 1920) and multiple intelligence theory Salovey and Mayer (1990), (Gardner, 1993), first proposed the concept of EI, which is “the ability to monitor one’s own and others’ feelings and emotions, to discriminate among them and to use this information to guide one’s thinking and actions (p. 5),” and defined it as a subset of social intelligence. EI was first introduced into management filed by Goleman (1995), who believed that EI is the ability to maintain self-control, enthusiasm and perseverance, and self-motivation, and that it consists of five major parts: (a) being aware of one’s emotions, (b) managing emotion, (c) motivating oneself, (d) identifying emotions in others, and (e) dealing with interpersonal relationships. The notion is quietly different from that of Salovey and Mayer (1990), who believed that EI is focused on the emotional ability to connect emotion with cognition.

There is a general agreement on the construction of models that divides EI into ability-based model and mixed model. The ability-based model of EI is focused on specific competence, and its core is emotion. The ability-based model was first proposed by Mayer and Salovey in 1997, which partially overlapped with cognitive ability. Mayer and Salovey (1997) defined EI as “the ability to perceive emotions, to access and generate emotions so as to help thought, to understand emotions and emotional knowledge, and to reflectively regulate emotions so as to promote emotional and intellectual growth (p. 5).” Based on the definition of EI by Mayer et al. (2008), MacCann et al. (2014) empirically verified EI as a second-stratum factor of intelligence and defined EI as the ability to process and reason emotional information, and that perception, understanding, and management of emotions are three dimensions of EI. Most scholars regard EI as a kind of ability, but Minbashian et al. (2017) conceptualized EI as a knowledge structure after analyzing measurement items of ability-based EI (Mayer and Salovey, 1997; Joseph and Newman, 2010). The knowledge structure specifically reflects an individual’s declarative knowledge of emotions, including knowledge of motivation and cognition of affective states, how emotions swing, how to form a more complex affective state, and strategies for regulating one’s emotions. In addition, EI is essential in social communication. Kidwell et al. (2011) introduced the concept of EI into marketing and defined EI as “the ability to acquire and apply knowledge from one’s emotions and those of others to produce beneficial outcomes (p. 78).” In this regard, Petrides and Furnham (2003) defined EI as a behavioral orientation related to an individual’s ability to recognize, process, and use emotional information, as well as self-cognition. Table 4 shows definitions of EI.

**TABLE 4:** Definitions of EI and its value.

References	Definition	Value
Salovey and Mayer, 1990	The ability to monitor one’s own and others’ feelings and emotions, to discriminate among them and to use this information to guide one’s thinking and actions.	The concept of emotional intelligence was first proposed and defined.
Goleman, 1995	The ability to control impulses, delay gratification, regulate moods, keep distress from obstructing cognitive functioning, and empathize.	Emotional intelligence was first introduced into management field and widely discussed in various fields.
Mayer and Salovey, 1997	The ability to perceive emotions, to access and generate emotions so as to help thought, to understand emotions and emotional knowledge, and to reflectively regulate emotions so as to promote emotional and intellectual growth.	The ability model of emotional intelligence was proposed firstly.
Bar-On, 1997	A set of non-cognitive capabilities and skills that influence one’s ability to succeed in coping with environmental demands and pressures.	A mixed model of emotional intelligence was first proposed.
Goleman, 1998	A synthesis of self-awareness, self-management, self-motivation, empathy and interpersonal skills.	The concept of emotional competence was first proposed and the emotional competence inventory (ECI) was developed.
Mayer et al., 2000	The ability to carry out accurate reasoning about emotions and the ability to use emotions and emotional knowledge to enhance thought.	A slight adjustment was made to the definition of Mayer and Salovey (1997) to develop the multifactorial emotional intelligence scale.
Petrides and Furnham, 2001, 2003	A constellation of emotion-related self-perceived abilities and dispositions, including individual differences in the ability to understand, process, and utilize affect-laden information.	The most comprehensive mixed model of EI.
Kidwell et al., 2011	The ability to acquire and apply knowledge from one’s emotions and those of others to produce beneficial outcomes.	The concept of emotional intelligence was first introduced into marketing exchange.
MacCann et al., 2014	The ability to process and reason affective information.	In the ability-based framework, the essence of emotional intelligence was empirically verified as a part of intelligence.
Minbashian et al., 2017	Including knowledge of the motivational and cognitive effects of various affective states, how emotions transition over time, how they combine to form more complex affective states, and strategies that can be used to regulate one’s affective states.	In the ability-based framework, the concept of emotional intelligence is extended to the concept of knowledge structure.

*Source: Collated according to relevant literature.*

### Dimensions and Measures of Emotional Intelligence

The dimension of EI is mainly based on two theoretical models: ability-based model and mixed model. Parallel to the theoretical model of EI is two measurement models, ability measurement and rating measurement. After distinguishing measurements based on the ability-based model and the mixed model, scholars have summarized three streams on measuring EI: (a) ability scales, (b) ratings of ability (self-reported), and (c) ratings of mixed model (self-reported or peer-reported) (Sackett et al., 2017; MacCann et al., 2020).

Mainstream research supports the ability-based model proposed by Mayer and Salovey (1997). This model divides EI into four dimensions: (a) perceiving emotions (the ability to identify and accurately express emotion), (b) emotions to facilitate thought (including not only using existing emotions to promote goal achievement but also generating new emotions in a particular situation to accomplish tasks), (c) understanding emotions (the ability to process emotional information), and (d) managing emotions (the ability to strengthen or weaken emotions). The measurement was called the Mayer-Salovey-Caruso Emotional Intelligence Test (MSCEIT), which is the most commonly used ability-based measure.

In the earliest ability-based model using self-report, EI consisted of the following three dimensions: perception of emotions, management of emotions, and emotional facilitation of thinking (Schutte et al., 1998). The measurement was the Assessing Emotions Scale (AES). However, Davies et al. (1998) proposed four dimensions: (a) one’s self emotional appraisal, (b) others’ emotional appraisal, (c) regulation of emotions, and (d) use of emotions. Subsequently, aiming at the four dimensions of EI, Wong and Law (2002) developed the Wong and Law Scale (WLEIS). In addition, in the Self-Rated Emotional Intelligence Scale (SREIS) developed by Brackett et al. (2006), EI is divided into four dimensions: perceiving emotions, using emotions, understanding emotions, and managing emotions.

In the mixed model, trait features are emphasized. Based on this view, there are many types of dimensions of EI, and major measurement scales include Emotional Competence Inventory (ECI) (Goleman, 1998), Emotional Quotient Inventory (EQ-i) (Bar-On, 2006), and Trait Emotional Intelligence Questionnaire (TEIQue) (Petrides et al., 2007). Goleman (1998) outlined that EI consists of four major competencies: self-awareness, self-management, social awareness, and social skills. Since Bar-On (2006) regarded the relationship between emotion and social function as the main content of EI in his study, EI consisted of five major domains, intrapersonal competence, interpersonal competence, stress management, adaptability, and general mood. Petrides et al. (2007) pointed out four dimensions of EI, happiness, emotion regulation, emotions, and relationships. Table 5 shows the dimensions and measurement of EI.

**TABLE 5:** Dimension and measurement of EI.

References	Model/Measures	Dimension	Content	Scale
Mayer et al., 2000, 2008	Ability model (ability scales)	Perceiving emotion	The ability to perceive of one’s and others’ emotion.	MSCEIT Scale
		Emotions to facilitate thought	The ability to using emotions to facilitate cognitive activities, such as thinking and problem solving.	
		Understanding emotion	The ability to understand verbal or non-verbal information.	
		Managing emotion	The ability to regulate emotions in oneself and others.	
Davies et al., 1998; Wong and Law, 2002	Ability model (self-report)	One’s self emotional appraisal	The ability to understand their deep emotions and be able to express these emotions naturally.	WLEIS Scale
		Others’ emotional appraisal	The ability to perceive and understand the emotions of those people around them.	
		Regulation of emotion	The ability to regulate their emotions and rapid recovery from psychological distress.	
		Use of emotion	The ability to use emotions toward constructive activities	
Schutte et al., 1998	Ability model (self-report)	Perception of emotion	Appraisal and expression of emotion in the self and appraisal of emotion in others.	AES Scale
		Management of emotion	Regulation of emotions in the self and regulation of emotions in others.	
		Emotional facilitation of thinking	Flexible planning, creative thinking, redirected attention and motivation.	
Jordan et al., 2002	Ability model (self-report)	Perceive own emotions	Recognize one’s own emotions.	WEIP Scale
		Discuss own emotions	Understand and assimilate one’s own emotions	
		Manage own emotions	Regulate and generate one’s own emotions.	
		Perceive others’ emotions	Recognize others’ emotions.	
		Manage others’ emotions	Empathize and manage others’ emotions.	
Brackett et al., 2006	Ability model (self-report)	Perceiving emotion	The ability to identify emotions in oneself and others, as well as in other stimuli.	SREIS Scale
		Using emotion	The ability to harness feelings	
		Understanding emotion	The ability to analyze emotions.	
		Managing emotion	The ability to reduce, enhance, or modify an emotional response in oneself and others, as well as the ability to experience a range of emotions.	
Goleman, 1998	Mixed model	Self-awareness	Accurate in one’s emotions.	ECI Scale
		Self-management	Control one’s emotions and behaviors.	
		Social awareness	Showing empathy to others, and having a service orientation and organizational awareness.	
		Social skills	Manage interpersonal relationships	
Bar-On, 2006	Mixed model	Intrapersonal competence	The ability to deal with internal emotions.	EQ-I Scale
		Interpersonal competence	The ability to deal with interpersonal emotions	
		Adaptability	The ability to deal with change flexibly.	
		Stress management	The ability to manage external pressure.	
		General mood	Description of general mood	
Petrides et al., 2007; Petrides, 2009	Mixed model	Happiness	More adaptable in general.	TEIQue Scale
		Emotion regulation	More willpower	
		Emotion	Egotism	
		Relationships	Interpersonal skills	

*Source: Collated according to relevant literature.*

## Influencing Mechanisms of Emotional Intelligence

In proving the rationality of EI, Salovey and Mayer (1990) pointed out that EI needs to process specific emotional information individually. Mayer and Salovey (1997) believed that EI is a group of abilities of processing emotional information. In fact, most studies generally believe that EI should be interpreted as “a specific ability to help individuals reason and use emotional information” (Fineman, 2006, p. 278; also see Mayer et al., 2000; Beck et al., 2001). This also means that the effectiveness of EI depends on the degree of effective recognition and utilization of emotional information.

**FIGURE 3:**
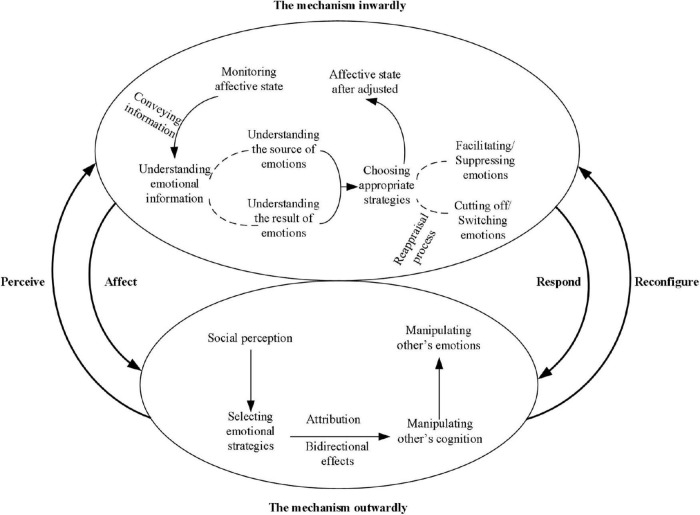
Two mechanisms of EI.

After analyzing the bodies of literature, we propose two specific mechanisms of how individuals with high EI use emotional information to influence themselves and others. The first mechanism involves processes that influence the self. Individual with high EI is good at using emotional cues to change their emotions so as to achieve goals such as reshaping their mental state, reducing stress, and improving the quality of decision-making (Bar-On, 2000; Kidwell et al., 2008). The second one is the mechanism by which emotional information is consciously released in social interactions to drive and screen the reaction of others. In the process, EI can not only be displayed publicly but also arouse the emotions of others. The two mechanisms are described in detail below (see Figure 3).

The internal mechanism refers to the process by which EI influences the self, and its trigger is the emotional fluctuation caused by events. Afterward, based on the abilities of emotion perception, emotion understanding, emotional promotion thinking, and emotion management described by Mayer et al. (2000), emotional information will undergo processing, which is the main content of this mechanism. Individuals with high EI tend to continuously monitor their emotional state (McCarthy et al., 2016). When emotions fluctuate, it is identified and transmitted as a set of unique emotional information (Izard, 1993). The understanding of this information not only lies in the emotion itself but also includes the source of emotion fluctuation and its consequences (MacCann et al., 2020). For example, when faced with unfair situations, job insecurity can trigger unpleasant emotions that stem from unfair behavior at work. In fact, people with low EI are more likely to engage in negative emotions than those with high EI, and it is difficult to find the root cause (Cheung et al., 2016; Dust et al., 2018). Both negative and positive emotions may result in potential threats or goal attainment (Lindebaum and Jordan, 2014; McFarland et al., 2016).

Based on sources and outcomes of emotions, individuals with high EI can use relevant knowledge to select the best strategy (Côté et al., 2010), such as promoting or suppressing emotions, cutting off or switching emotions after reappraisal to maintain a good emotional state. Specifically, if the desired outcome is of help to individual goals, emotional management capability will help individuals to maintain or reinforce existing emotional states, thereby inducing or enhancing individual behavior or motivation (Joseph and Newman, 2010). On the contrary, when existing emotions may lead to negative results, individuals with high EI may suppress current emotions or reappraise their emotional sources. Emotion suppression is a reasonable strategy when excessive emotion can lead to negative results. For example, individuals with high EI may suppress their emotions when they find that self-perceived emotions are too optimistic and may lead to wrong decisions (Lerner and Keltner, 2001; Côté et al., 2010). There are two steps to reappraise the source of emotion. First, individuals should think about whether the source of the emotion is worthy of attention, which ensures that reasonable concerns are proactively addressed and irrelevant distractions are ignored (Dust et al., 2018). Once the emotional source can affect task performance, individuals with high EI will no longer consider the information related to this source, which helps to cut off the existing adverse emotion to restore the desired emotional state. Second, when the emotional source needs to be paid attention, individuals with high EI will reappraise their cognition of the emotional source. For example, when there is anxiety, tension, and other negative emotions due to work pressure, employees with high EI will regard stressful tasks as challenges rather than threats, thus arousing work enthusiasm (Dong et al., 2014), which also means emotional transformation. In general, individuals with high EI can continuously monitor their emotional states, identify and analyze the sources and results of emotional cues, and then adopt beneficial strategies to achieve desired emotional states and expected goals.

Social perception influences the process by which individual EI is associated with responses of others (Chowdry and Newcomb, 1952; Côté et al., 2010). Individuals with high EI can not only accurately identify others’ emotions and their sources but also have a strong perception of subtle changes in emotional atmosphere as well as the direction or reason of such changes, which can help them acquire a lot of knowledge of emotional cues. This knowledge enables them to adopt more appropriate emotional strategies by identifying, understanding, and processing needs from the other person (George, 2000; Wolff et al., 2002). Then, individuals with high EI will select emotional strategies, such as promoting, suppressing, inducing, and faking emotions to display their emotional information to their counterparts by adopting reasonable emotional strategies.

In the context of interaction, attribution is an indispensable step in the process of emotional expression (Eberly and Fong, 2013). When others receive emotional information displayed by individuals, they will explain and analyze the emotion through the attribution process, including the source of the emotion and the motivation and intention of individuals with this emotional information (Dasborough and Ashkanasy, 2002). This means that individuals with high EI may be able to use appropriate strategies to only express emotions that others wish to perceive and interpret (Humphrey et al., 2008). This kind of emotional information can affect others’ cognition of the expressor’s personal characteristics or behavior. For example, a leaders’ emotional display of enthusiasm and concern when communicating and motivating members also encourages members to attribute this to the leader’s motivation to try to implement transformational leadership (Dasborough and Ashkanasy, 2002). In addition, people with high EI are able to manipulate other people’s emotional response by directing their cognitive processes.

It is important that in social interactions, the effect of EI may not be one-way but bidirectional. Referring to the emotional and intentionality attribution model in leader-member relationship proposed by Dasborough and Ashkanasy (2002), EI can influence intentionality attribution in a social context. Specifically, for a person who exhibits emotional cues, the emotional strategies he uses and the extent to which he uses them may influence the attributions of another person. However, individuals with high EI can accurately perceive and interpret the intention of another party, thus producing the related emotional response. In addition, individuals with high EI may manipulate the cognitive attribution and emotion of another party to effectively promote the generation and reinforcement of the other party’s motivation or behavior, thus affecting their performance (Humphrey et al., 2008).

## Framework Development of Emotional Intelligence Research

**FIGURE 4:**
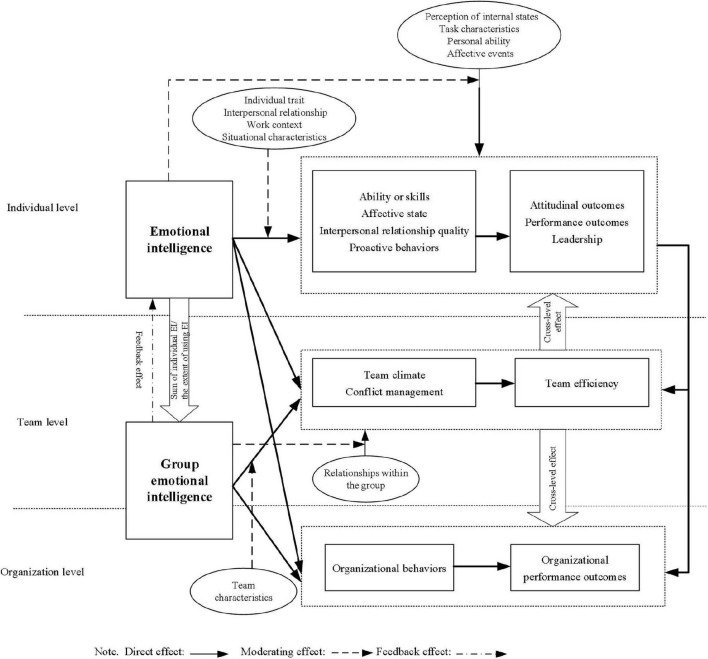
Theoretical framework of EI.

In order to comprehensively review the development of EI and provide insight for future research, a theoretical framework is developed (see Figure 4) in this study. This framework is based on the following parts: (a) notion of EI (individual and team EI); (b) outcomes of EI; (c) moderating factors that regulate the relationship between EI and its outcomes; (d) moderating effect of EI. As the concept of EI is controversial (Elfenbein, 2008; Joseph and Newman, 2010), the ability-based model is widely regarded as the most suitable model for EI (Jordan et al., 2003), so we reviewed EI from the perspective of the ability-based model.

### Individual Emotional Intelligence

Mayer and Salovey (1997) proposed the ability-based model of EI, which covers a variety of abilities to understand and process emotional information, including emotional perception, emotional promotion, emotional understanding, and emotional regulation. This is a generally agreed theoretical model describing the composition of EI capabilities (Kidwell et al., 2008; MacCann et al., 2020). Since each can act independently or in combination with each other, Mayer and Salovey (1997), based on this model, argued that the above four aspects are progressive (Mayer and Salovey, 1997; Brackett et al., 2006; Joseph and Newman, 2010). Specifically, emotional perception as the ability to perceive emotions in oneself and others precedes other capabilities. Emotional promotion helps individuals promote cognitive activities using perceived emotions. Since emotion understanding requires language skills and logical thinking, the cognitive enhancement brought by emotion promotion can be directly reflected in emotion understanding. Emotion regulation relies on the processing and analysis of emotional information in emotion understanding (Mayer and Salovey, 1997).

### Group Emotional Intelligence

When a high level of interpersonal interaction and emotional cues arise in teams, the influence of individual EI can be reflected at the team level through the activated interpersonal communication mechanism, and the aggregation of individual EI will form a team-level construct called group emotional intelligence (GEI, also known as collective emotional intelligence) (Farh et al., 2012; Troth et al., 2012; Wang, 2015). GEI is first proposed by Druskat and Wolff (2001) and is defined as “the ability of a group to develop a set of norms that manage emotional processes” (p. 132). These norms encourage the expression and regulation of emotional dynamics within and outside a group, thus helping group members to deal with emotional problems more effectively (Curseu et al., 2015). Extant studies on the formation of GEI mainly fall into two streams: one believes that GEI is the sum of individual EI resources (Jordan and Troth, 2004), and the other believes that GEI is the extent to which groups use EI when communicating with each other (Elfenbein, 2006). The former is focused on personal resources brought by members, while the latter is focused on interaction between members (Curseu et al., 2015). Since teams with high EI are better able to “interpret” emotional information and respond to different emotional situations, most studies believe that GEI includes group emotional awareness and group emotional regulation (Jordan and Lawrence, 2009; Troth et al., 2012).

### Outcomes of Emotional Intelligence

Emotional intelligence can directly or indirectly affect results through mediating variables. Considering that EI as an ability to use emotions provides a potential emotional background for most of our behaviors and ongoing thought processes (Forgas and George, 2001), and its influence on behavior at different levels is quite profound. Therefore, we reviewed the effect of EI on individual, team, and organizational outcomes.

#### Outcomes at Individual Level

As shown above, individual EI can directly affect abilities or skills, affective state, interpersonal relationship quality, and proactive behaviors at individual level.

##### Abilities or Skills

Individual EI can be of help to improve skills in interaction with others. The perception, understanding, and regulation of emotions are conducive to the ability to adapt to environment and stress in organizations. In addition, emotion promotion can help employees perceive problems from multiple perspectives, which may make them more willing to consider and even seek opinions from others, thus improving self-cognition and skills (Sheldon et al., 2014). Besides, EI enables individuals to have a keen understanding of interpersonal dynamics. On the one hand, it enables individuals to adjust their emotions to the environment more quickly, which helps to strengthen individual interpersonal skills and improve social and political skills (Zaccaro et al., 2018). On the other hand, it encourages individuals to more accurately identify the behavioral connotations of interactions. Dasborough and Ashkanasy (2002) believed that EI could enable individuals to more accurately perceive and interpret emotional cues, assess and classify others’ motivations more accurately, and, thus, enhance their ability to perceive others’ intentions.

##### Affective State

Individuals with high EI have a better understanding of how to regulate their emotions to achieve a favorable emotional state. Geddes and Callister (2007) pointed out after exploring emotional expression in the workplace that employees with high EI are more likely to deal with anger rationally. Emotional understanding and regulation can also speed up members’ recovery from negative emotions (Shepherd, 2009) and induce employees to experience positive emotional states (Parke et al., 2015). In addition, objective appraisal and emotional knowledge encourage individuals to reappraise and regulate their emotions in order to achieve and maintain a stable emotional state (Kidwell et al., 2008; Dust et al., 2018).

##### Interpersonal Relationship Quality

Most studies support the positive relationship between EI and interpersonal relationship quality (Kellett et al., 2006; Kotsou et al., 2011; Yela Aránega et al., 2020). An individual with high EI is very keen to non-verbal cues and can accurately catch the emotions of others and needs not clarified, and provide appropriate help to regulate the emotional responses of other parties, so as to achieve the purpose of strengthening relationship quality, such as establishing trust, or enhance interpersonal effectiveness (Chun et al., 2010; Farh et al., 2012; Momm et al., 2014; Vidyarthi et al., 2014).

##### Proactive Behaviors

Because of effective emotion regulation, members with high EI are more likely to exhibit positive behaviors than those with low EI (Kim et al., 2005). Studies argued that individuals with low EI have a weak ability in emotional awareness and emotion management, so they feel that it is more difficult to deal with the consequences of negative emotions compared with individuals with high EI (Jordan et al., 2002). Even when facing pressure, they tend to adopt more negative coping strategies (Kim et al., 2009). Conversely, individuals with high EI are able to interpret interpersonal behavior and subtle emotional cues more accurately, which means that the benefits of taking the initiative may far outweigh the cost of personal resources if they are able to solve their problems through social interaction. Therefore, motivated by resource conservation, they are more likely to engage in proactive behaviors, such as advising leaders, seeking feedback, or developing relationships with supervisors (Kim et al., 2009; Grant, 2013; Halbesleben et al., 2014). In addition, in order to develop social relationships, individuals with high EI may also adopt positive behaviors related to emotions, such as using humor to manage conflicts, adopting emotional labor strategies to achieve higher performance, and self-monitoring to establish relationships with others (Romero and Pescosolido, 2008; Caldwell, 2009; Cheung and Tang, 2009). Besides, as individuals with high EI tend to regard others’ sadness and anxiety as a signal to seek help, they tend to carry out an organizational citizenship behavior (Kluemper et al., 2013).

In addition to the above direct influence of EI, individual EI can also indirectly affect subjective attitude, performance outcomes, and leadership.

##### Attitudinal Outcome

Studies have shown that individual EI is correlated with work attitude outcomes, including job satisfaction, organizational commitment, and personal well-being (Wong and Law, 2002; Pillai and Krishnakumar, 2019). These attitude outcomes may be self-relevant or related with others (Wong and Law, 2002; Chiva and Alegre, 2008). The organizational members’ keen perception of emotional cues and reaction-centered emotional management ability will enable them to build stronger interpersonal relationships and maintain continuous positive emotional states, which will both help improve their job satisfaction and enhance their emotional commitment to an organization (Wong and Law, 2002; Dong et al., 2014). Chiva and Alegre (2008) believed that individual EI would have a positive impact on organizational learning ability, thus improving job satisfaction. Furthermore, emotional commitment to others is a necessary component of social interaction (Ashkanasy and Hooper, 1999). According to social exchange theory, members of an organization can respond to other’s emotional states or behavioral information accordingly. In this sense, members with high EI can obtain desired attitudinal results by satisfying the psychological needs of other parties. For example, Wong and Law (2002) pointed out that leaders with high EI were more inclined to maintain employees’ positive emotional states, which positively affected subordinates’ happiness and job satisfaction.

##### Performance Outcomes

More and more studies have proved the positive relationship between EI and performance outcomes (Zhou and George, 2003; Vidyarthi et al., 2014; Deming, 2017), which include task performance, decision quality, creativity, and productivity. Specifically, members with high EI are good at regulating their emotions and are more likely to acquire a great deal of knowledge of how to use emotions to achieve their goals, such as participating in brainstorming in a passionate or excited mood (Gohm and Clore, 2002; Parke et al., 2015), which, in turn, is conducive to improve employees’ creativity and job performance, and help members maintain a favorable emotional state. Even if they are having negative emotions, members with high EI will face the source of negative emotions and control their emotions within an appropriate threshold to cultivate more acute awareness and make wise decisions (Gohm et al., 2005). In addition, Kidwell et al. (2008) pointed out in their empirical study that individuals with high EI can maintain stable emotional states through emotional regulation, thus improving the quality of decision-making.

Because of understanding of the source of emotions, individuals with high EI are more likely to be employed in organizations that match their values (Tugade and Fredrickson, 2007; Dust et al., 2018), which means that they may be more proactive after being employed, such as taking a positive voice behavior (Grant, 2013), which helps improve their work performance. In a social context, the improvement of social skills brought by EI contributes to the exchange of heterogeneous resources and information during interaction, which will improve the efficiency of members (Kim et al., 2009; Deming, 2017). In the same context, individuals with high EI also influence others’ emotional states or behavioral tendencies through exchange of social emotional resources, thus affecting others’ job performance (Vidyarthi et al., 2014). For example, Zhou and George (2003) believed that leaders with high EI can accurately perceive employees’ frustration at work and encourage employees to cultivate positive emotional states, thus promoting the generation of high-quality ideas and improving employees’ work efficiency.

##### Leadership

Studies have confirmed the positive relationship between EI and leadership. Leadership refers to “a process of social interaction where leaders attempt to influence the behavior of their followers” (Yukl et al., 2002, p. 615). When exploring the above, Walter et al. (2012) believed that individuals with high EI might be better at using emotional information to coordinate team tasks in persuasive ways, thus demonstrating their leadership. In general, current studies are mainly focused on the influence of EI on leadership effectiveness and different types of leadership, such as transformational leadership, charismatic leadership, and relationship-oriented leadership (Brown and Moshavi, 2005; Cavazotte et al., 2012).

To be specific, leaders with high EI are good at manipulating employees’ perceptions with high-quality vision statements to encourage employees’ commitment to a vision, thus improving the effectiveness of transformational leadership (Hur et al., 2011). Leaders with high EI can strengthen charismatic leadership by maintaining a positive attitude and effectively using impression management strategies (Walter and Bruch, 2009). Recognizing and regulating others’ emotions can also promote the exchange of emotional resources between each other, which is conducive to the development of relational-oriented leadership (Kellett et al., 2006). In addition, George (2000) believed that leaders with high EI can improve their leadership efficiency by setting collective goals, instilling awareness in employees, maintaining enthusiasm, and strengthening trust. Rosette and Ciarrochi (2005) also empirically confirmed the positive correlation between leader’s EI and leadership effectiveness.

#### Outcomes at Team Level

Both individual EI and GEI have an impact on team level outcomes. For the former, leaders’ EI can influence internal process and team performance at team level. For the latter, GEI helps teams understand and respond to members’ emotional responses through normative and shared behavioral patterns, and influences team effectiveness through mutual trust and group identification (Yang and Mossholder, 2004; Lee and Wong, 2017). In general, current studies show that team climate and conflict management are considered to be results of EI at the team level, and that EI may have an indirect impact on team efficiency.

##### Team Climate

Team climate is the emotional atmosphere that affects employees’ emotional expression and experience. Emotions are often recognized as drivers of behavior and ultimately affect employee performance (Ashkanasy et al., 2017). Pirola-Merlo et al. (2002) believed that leader’s EI has a strong influence on the formation of team climate. Leaders with high EI can better identify members’ emotional needs and consciously manage their emotions (Eberly and Fong, 2013). Emotional expressions of a leader also affect subordinates. Therefore, leaders can create a positive emotional climate by setting good examples (Humphrey et al., 2015) or suppressing negative emotions. For example, leaders suppress self-doubt in the face of adversity in order to express positive emotions to team members (Hur et al., 2011). Wilderom et al. (2015) also believed that leaders with high EI can create or maintain a cohesive atmosphere in a team by stimulating positive group identity, establishing group norms, or encouraging team members to participate in emotional expression.

##### Conflict Management

It is obvious that conflict is a reflection of internal emotions in a team (Jordan and Troth, 2004). Individual or group EI may affect a conflict within a team. Leaders with high EI have a stronger perception of emotional information in a team and are able to catch conflict and tension in the team. Therefore, they may be better at correctly using the social influence brought about by power distance to effectively coordinate conflicts between members (Castro et al., 2012). In addition, because high-EI leaders can more accurately understand their inner emotions and needs, they can also set workplace norms accepted by a group, thus reducing the occurrence of team conflict and maintaining a harmonious atmosphere within a team (Wilderom et al., 2015).

Current studies show that teams with high GEI are better at managing conflict than teams with low GEI (Druskat and Wolff, 2001; Jordan et al., 2002; Jordan and Troth, 2004). Specifically, because individuals have different ideas about team tasks, team members may have perceived threats, which may lead to adverse emotional conflicts. Teams with high EI resolve these differences through open discussion and collaboration among members (Jordan and Troth, 2004). In addition, teams with high GEI can develop a set of norms to support emotional regulation to better identify conflicts in a timely manner and find different creative solutions to avoid escalation of conflicts (Yang and Mossholder, 2004; Curseu et al., 2015). For example, Lee and Wong (2017) suggested that GEI can manage task conflicts and relationship conflicts in a collaborative manner and prevent task conflicts from transforming into relationship conflicts (Curseu et al., 2015).

##### Team Efficiency

Druskat and Wolff (2001) proposed a team effectiveness model in the study of GEI. In this model, GEI contributes to better decisions, more creative solutions, and higher productivity. Indeed, in line with the suggestions put forward by Druskat and Wolff (2001), numerous studies have examined empirically the link between GEI and team effectiveness, and believed that there is a positive relationship between GEI and team efficiency (Hur et al., 2011; Curseu et al., 2015; Lee and Wong, 2017; Jamshed and Majeed, 2019). In order to effectively deal with emotional challenges, leader’s or group’s EI will assist teams to build a positive team climate and encourage proactive solving of intra-team problems; the latter is mainly manifested in the coordination of conflicts (Druskat and Wolff, 2001; Pirola-Merlo et al., 2002). Specifically, leaders or groups with high EI create an emotional atmosphere that enables members to perceive the information expected by an organization and generate corresponding emotions or motivations. For example, an open and cooperative atmosphere promotes the emergence and proliferation of new ideas. Under these circumstances, emotional contagion among members will be of help to improve team performance and enhance team creativity (Hur et al., 2011). In addition, teams and leaders with high EI are good at coordinating intra-team conflicts, reducing spread of adverse emotions, and keeping task conflicts within a favorable threshold. A moderate tension within a team is conducive to promoting team creativity and improving team effectiveness (Jamshed and Majeed, 2019).

#### Outcomes at Organization Level

The influence of EI on the organizational outcomes is mainly in three ways, namely, entrepreneur’s EI, top management teams ‘EI and high EI team affect organizational behavior respectively, thus affecting organizational performance.

Upper echelons theory holds that entrepreneurs and top management teams have a great effect on strategic behavior and performance (Hambrick and Mason, 1984). Facing high risks and uncertain environments, entrepreneurs tend to be emotional when making decisions (Cardon et al., 2012). Since entrepreneurs may have preferences or beliefs such as loss aversion and overconfidence, an entrepreneur’s bias against alternative options may reduce the effectiveness of decision-making. Entrepreneurs with high EI have a broader vision and sharper insight, can objectively evaluate their behavior and market response, reduce bias, and coordinate different cognitive processes to improve decision-making quality and adaptability (Azouzi and Jarboui, 2014). Blume and Covin (2011) also pointed out that entrepreneurs with high EI have stronger intuitive judgment ability. This also means that entrepreneurs with high EI are more likely to develop effective strategies. In addition, entrepreneurs with high EI are able to more convincingly present their vision to employees and effectively manage social networks, which not only help them build and maintain trust with stakeholders but also gain access to information and resources (Azouzi and Jarboui, 2014; Naude et al., 2014).

Influencing factors of strategic decision quality of top management teams are mainly divided into two categories: one is the characteristics of senior management team, including GEI, and the other is the operation process of top management teams, including communication and coordination within a team (Hambrick and Mason, 1984). As one of the characteristics of top management teams, GEI can reduce the negative impact of emotional biases on decision-making by developing emotional norms and maintaining the dynamic stability of team emotions. Besides, GEI can also affect the operation process of top management teams, such as by enhancing intra-team communication, accurately interpreting intra-team information, and carrying out high-quality feedback loops to enhance decision-making quality (Wang, 2015).

### Moderators in Emotional Intelligence Research

In recent years, more and more studies have revealed boundary conditions under which EI plays a role. It is found that moderators affect the relationship between EI and its outcomes at the individual and team levels. At the individual level, individual characteristics (including behavior tendency and trait motivation), interpersonal relationship, work background, and situational characteristics (including others’ EI and GEI) may moderate relationships between individual EI and its outcomes (such as skills, emotional state, relationship quality, and proactive behavior). At the team level, team characteristics are considered to be the moderating variable of the relationship between team EI and team atmosphere and conflict (Shepherd, 2009; Wang, 2015).

#### Moderators at the Individual Level

##### Individual Trait

Individual differences may influence the relationship between EI and work outcomes (Côté and Miners, 2006; Rode et al., 2007). As the main source of individual differences, trait characteristics can affect individual behavioral tendencies or trait motivations (Wright et al., 1995; Petrides, 2009). For individuals with behavioral tendencies, high EI is more likely to lead to active behaviors. For example, Walter et al. (2012) believed that EI is more likely to trigger visible behaviors for individuals with high extroversion. Driven by a motivation, EI may have a great influence on expected results. For example, conscientiousness is seen as a motivation to achieve goals. Members with high conscientiousness are more likely to enhance the ability of EI to get rid of negative emotions, thus improving job performance (Rode et al., 2007).

##### Interpersonal Relationships

Empirical studies have shown that interpersonal factors can moderate the relationship between individual EI and others’ behavior (Vidyarthi et al., 2014). This interpersonal relationship has been described as psychological distance from others; the stronger the relationship, the closer the psychological distance. In the interaction between leaders and employees, the high-power distance caused by difference in rank reflects the long psychological distance between leaders and employees. This psychological distance hinders the flow of social emotional resources in the interaction process, resulting in the inability to get corresponding feedback in the transmission of emotional information and limitation in the impact of leaders’ emotional perception on employee behavior (Offermann and Hellmann, 1997; Vidyarthi et al., 2014; Pillai and Krishnakumar, 2019). Information theory holds that the understanding of emotional information is valuable. In highly intimate relationships, individuals with high EI perceive others’ emotional information more quickly and accurately, and are more easily affected by others’ emotional information. Pillai and Krishnakumar (2019) also pointed out that individuals with high EI are more likely to engage in positive social behaviors under the condition of high relationship intimacy, which will further improve their own happiness after receiving emotional feedback from another party.

##### Work Context

A large number of studies have found that work contexts, such as emotional labor and social needs, moderate the relationship between EI and work outcomes (Elfenbein and Ambady, 2002; Wong and Law, 2002; Vidyarthi et al., 2014). Empirical studies show that compared with low emotional labor, a work background with high emotional labor is more likely to contain emotional cues and social information, which will effectively activate individual EI (Ybarra et al., 2014). When individuals rely on others (colleagues or team leaders) and obtain support from others to complete their work with high task dependence, individual emotion recognition and emotion management will bring smoother interpersonal communication, thus affecting individual work results (Elfenbein and Ambady, 2002). When exploring the relationship between EI and job performance, Farh et al. (2012) also pointed out that EI is more strongly correlated with job performance in a work context with high management requirements.

##### Situational Characteristics

Situational characteristics (which refer to the EI of another party in an interaction, or GEI) can influence the effect of EI. The interaction between individual EI and others’ EI may produce complementary effects (Chun et al., 2012). The effect of EI is also affected by situational factors, and that is the EI of the other party in the interaction, or the EI of the group. Chun et al. (2010) argued that both parties with high EI have a better understanding of each other’s emotions and needs, so as to timely regulate their emotional responses and promote the development of high-quality relationships such as trust. If one party has low EI, the other party with high EI can also enhance the quality of the relationship through a complementary effect. In addition, with high GEI, teams can handle negative emotions and find resources to deal with emotional recovery (Shepherd, 2009), which will strengthen the positive effect of EI of team members.

#### Moderators at the Team Level

Boundary conditions affecting the relationship between GEI and team-level outcomes are mainly related to team characteristics, such as number of individuals with high EI in teams and team informational diversity. Shepherd (2009) suggested in a multi- and meso-level model that the positive relationship between GEI and its speed to recover from adverse events is enhanced for teams with more emotionally intelligent members. Teams with high GEI store norms that direct members to fulfill different roles (Sameroff, 1994), while behaviors of members with high EI may change or enrich team norms and behavior patterns, which will enhance the influence of GEI (Schoka Traylor et al., 2003). In addition, these norms developed by GEI help teams to efficiently and intelligently process emotions, maintain emotionally stable communication (Druskat and Wolff, 2001); it also means that in a need of communication situations, for example, under the situation of diversity information being exchanged continually among members, there is a stronger link between EI and performance (Wang, 2015).

### Moderating Effects of Emotional Intelligence

There have been studies that have used EI as a moderator in the organization field. Individual EI and GEI could moderate these relationships between predictors and work outcomes at the individual and team levels, respectively. What follows is a brief description of moderating effects of EI in different contexts.

#### The Moderating Effect of Emotional Intelligence at Individual Level

When EI operates as a moderator, it mainly moderates the relationship among internal state perception, personal ability, task characteristics, and emotional events on individual behaviors and work outcomes.

##### Internal State Perception

According to self-consistency theory (Korman, 1967; Dust et al., 2018), internal states, such as workplace anxiety, stress, and emotional state, will directly affect behaviors or work outcomes. Negative internal states can lead to negative outcomes, and EI, as the ability to monitor and manage emotional cues and facilitate emotional shift, is considered a promising source for regulating emotion (Di Fabio and Saklofske, 2021). Many scholars have proposed the importance of EI in coordinating internal states and emotional responses or behaviors (Cheng and McCarthy, 2018; Di Fabio and Kenny, 2019). Jordan et al. (2002) pointed out that members with high EI could not completely avoid the negative emotions associated with job insecurity, but that they could break the relationship between job insecurity and negative behaviors. Cheng and McCarthy (2018) also believed that members in an anxious state but with high EI are better able to understand the extent to which concerns affected their concentration and shifted their attention from the distraction to a current task. In addition, because high-EI members have more accurate emotional knowledge base and can better understand how their emotions affect their thought process and environmental perception, when they perceive negative situations such as threats or stress, they can also reduce negative effects through reappraisal (McFarland et al., 2016).

##### Personal Ability

Emotional intelligence can moderate the relationship between individual abilities and work outcomes. Obviously, EI and cognitive intelligence have an independent and complementary influence on job performance. When an individual’s cognitive intelligence is low, EI can fill the gap by acquiring goal-related information, strengthening the quality of social relationships, and enhancing the quality of motivation and decision-making, thus positively affecting the relationship between cognitive ability and job performance. EI can also strengthen the influence of organization members on customers, because high-EI members can more accurately know customer needs and exert influence with the best solution (Kidwell et al., 2011). When EI is low, interpersonal skills do not work.

##### Task Characteristics

Transactional theory of stress and coping (TTSC) argues that stressful jobs are regarded as challenges or threats and lead to different emotional experiences that affect productivity (Lazarus and Folkman, 1984, 1987). This theory explains the moderating effect of EI on the relationship between task characteristics and performance. Members with high EI are more sensitive to emotional experiences at work (Clercq et al., 2014). For tasks with multiple attributes, members with high EI can effectively reappraise these tasks and convert them into motivation or opportunities, thus completing tasks more effectively (Day and Carroll, 2004). For instance, Clercq et al. (2014) proposed that employees with high EI are better able to recognize the value of goal congruence and use EI when exploring the relationship between goal congruence and organizational deviation. Such positive emotional information embedded in their relationship can reduce their possible organizational biases. Members with high EI can also reevaluate development opportunities to eliminate negative impacts (Dong et al., 2014). Parke et al. (2015) also confirmed that EI can transform information and organizational requirements into internal incentives, thus improving employees’ creativity (Thory, 2013).

##### Affective Events

Affective events theory proposes that accumulation of emotional events influences an individual’s emotional state, which in turn influences the individual’s attitude and behavior (Weiss and Cropanzano, 1996; Herman et al., 2018). As an effective theory to explain emotional fluctuations, this theory can explain the regulatory role of EI in emotional events. When an event is internally stress-induced, EI affects an individual’s ability significantly to process the event (Dust et al., 2018). Lebel (2017) believes that EI can help individuals focus on future actions rather than events that trigger emotions. When facing fears, members can take advantage of e emotions, constructively guide fear emotions, and strive to seek feedback or take positive actions. When facing failure, managers with high EI may continuously face failure without hesitation, which will let them know their own capabilities (Sheldon et al., 2014). For managers with low EI, when they face negative feedback, the concern of self-protection may lead to the motivation of avoiding feedback, so they cannot timely respond fast.

#### The Moderating Effect of Emotional Intelligence at Team Level

Intra-group relationships not only reflect the quality of social interaction and emotional experiences in a team but also convey the social emotional information related to intra-group conflicts (Lawler, 2001; Yang and Mossholder, 2004). Since group norms developed by GEI can reflect the expected emotional tone of behavior during a conflict, GEI has been considered as a boundary condition to explain the relationship between intra-team conflicts and team effectiveness in many studies (e.g., Yang and Mossholder, 2004; Ambrosini et al., 2007; Lee and Wong, 2017). Yang and Mossholder (2004), in exploring the role of EI in the process of intra-team conflicts, indicated that teams with high GEI can keep task conflicts from spreading to an interpersonal relationship, that is, reduce links between task conflicts and relationship conflicts. In addition, GEI can effectively reduce the adverse effects of relationship conflicts and weaken the negative relationship between relationship conflicts and team performance (Koman and Wolff, 2008). Speaking of negative effects of conflicts, Ayoko et al. (2008) also showed empirically that GEI can moderate the relationship between conflicts and destructive reactions to a conflict, that, when GEI is high, the negative effects weaken. Relationships within a group are reflected not only in intra-team conflicts, but also in group affective tones. Collins et al. (2015) provided some evidence to show that a positive emotional tone positively predicts team performance when GEI is high; otherwise, it’s a negative predictor.

Based on the above analysis, we propose a theoretical framework of EI in an organization, as shown in Figure 4. In this model, the following three aspects need to be noted: first, not all facets of EI exert an equal and consistent influence on outcomes, and the sub-dimension of EI might exert an influence independently as a single factor. Second, EI in organizations can have a cross-level effect. For example, when organizational members have a high degree of control in a team or an organization, they can influence the quality of decision-making by improving their EI, thus indirectly affecting team efficacy and organizational effectiveness. The enhancement of team effectiveness driven by GEI can also affect organizational performance. For example, a top management team will be directly affected in an organization. The improvement of the effectiveness of multiple teams with high GEI can promote organizational efficiency. In addition, a team climate created by GEI can also drive individual behaviors or emotional states across levels. Third, the feedback effect emerges in the developing process of EI. The construction of GEI relies on the aggregation of individual EI in the interaction, in turn, EI of members in emotionally intelligent teams and can be developed by emotional training or cultivated through organizational culture (Kaplan et al., 2014; Kidwell et al., 2015).

## Discussion and Conclusion

On the basis of visualized analysis and mechanism analysis, we mainly draw the following conclusions: (1) the results of visualization show that EI has been gradually paid attention by many scholars, and that the number of articles published has been increasing; (2) the influencing mechanism of EI mainly includes self-effect mechanism and influencing others mechanism. The influence chain of “internal state monitoring – emotional information understanding – optimal strategy selection – emotional state” is adopted in the self-effect mechanism. The influencing others mechanism means that individuals manipulate others’ cognition and emotion through an attribution process after external perception and application of emotional strategies; (3) EI can be divided into two categories, individual EI and group EI, and its effects are mainly at the level of individuals, teams, and organizations. Compared with the multiple effects of EI on the individual level, the team level mainly focuses on the influence of EI on the internal atmosphere or a team conflict, while the organization level mostly explores the influence of EI under the upper echelon theory. It is obvious that there are few perspectives and narrow research scope at the team and organization levels; (4) EI is mainly regarded as an independent variable or a moderating variable, and hardly as a mediator variable. It goes without saying that EI has attracted more attention from scholars, but that there are still many valuable issues that need to be paid attention in the future. Therefore, we propose possible future research directions.

First, future research can validate a more appropriate EI measurement scale. Based on the ability-based model and mixed model, current studies have proposed complex measures of EI. However, some scholars still hold critical opinions on the measurement scale of EI. Future research can supplement or revise the EI scale from the following aspects. First of all, because the concepts of EI overlap with traditional personality factors (O’Connor and Little, 2003), scholars believe that it lacks convergent and discriminant validity (Mayer et al., 2008; Joseph and Newman, 2010). The problem of validity can be solved in the future. Besides, self-reported surveys are susceptible to social expectations, and respondents may falsely report the results. In this regard, scholars can improve the language of measurement items to reduce emotional bias or adopt group measurement and use reverse questions in the items to avoid self-reported concerns. Finally, the validity of measures of EI is called into question, because the same level of EI may trigger different behaviors in different cultures. Therefore, cultural factors can be considered in the measurement scale of EI in future studies.

Second, EI, as an important factor at multiple levels in an organization, is focused on the individual level from the perspective of social capital or social network (Momm et al., 2014; Naude et al., 2014). Seldom attention is given to the team and organizational levels. Team level studies mainly emphasize changes in the internal state of teams from the perspective of communication, while those at the organizational level mostly analyze it from the perspective of managers’ emotions (Curseu et al., 2015). These above perspectives limit potential EI research. Therefore, future research should focus on antecedents of EI and check under what circumstances individual or group EI can be activated to trigger or promote corresponding behaviors. Furthermore, it is essential to track the development of EI from a dynamic perspective. Since EI can be improved through training, dynamic research on the relationship between EI and its results is helpful to characterize the specific influence of EI and explore the long-term influence mechanism of EI on organizational outcomes, which means that cross-level research on EI is of importance in the near future, and that more attention should be paid to group EI’s influence on the organizational level.

Third, future research should deepen the comparative research on EI and its different dimensions. Researchers found that the effect of EI with different constructs may be quite different (Côté and Miners, 2006; O’Boyle et al., 2011) because of a discordant concept of EI. In addition, many scholars no longer explore EI as a whole variable but to study different influences of a single dimension of EI, for example, emotion regulation (Walter et al., 2012; Momm et al., 2014). However, the dimensions of EI are not completely independent of each other. Future research should explore and compare the effects of different types of EI on multiple levels. In addition, it is also meaningful to compare the influence of each dimension under different backgrounds.

Fourth, future research should focus on bidirectional effects between EI and its outcomes. Most studies argue that EI is a born factor or the result of professional training (Kaplan et al., 2014; Kidwell et al., 2015). However, EI is rooted in a special context, and the bidirectional effects of EI and its outcomes on each other are very complicated. When EI influences subsequent outcomes, it may also affect EI through feedback and recursive process. The influencing process should be longitudinal, in which we can not only address the influence of EI on its outcomes but also resolve the concerns of the effects of these outcomes on EI. The feedback path can give explanation to the bidirectional effects. Current studies on the measurement of EI use cross-section data (Jordan and Troth, 2004; Kim et al., 2009) and cannot show the longitudinal feature of EI. Future studies should focus on longitudinal designs to observe the fluctuations of EI, which will be of help to test the dynamic influence of and on EI.

Finally, future research should focus more on negative effects of EI, which may be interesting. Most studies believed that EI has a positive impact on all levels of an organization. However, other research suggests that EI may not be generally good. Higher EI may drive people to know too much about situations and respond to interfering emotional cues (Elfenbein and Ambady, 2002; Farh et al., 2012). Although scholars have pointed out possible adverse effects of EI, there is still lack of empirical studies on it. We suggest that future research should put more emphasis on the negative role of EI; one is to explore the negative relationship between EI and its outcomes, and, for example, when employees are full of emotional information, while its working performance is less related to social communication, so that employees with high EI may bring negative performance outcomes. Besides, considering that there may be multiple thresholds between EI and its outcomes, whether there is a non-linear effect is also worth further exploring, for example, during an interaction with leaders, appropriate EI displayed by employees can lead to positive feedback from leaders; whereas when leaders find that employees’ EI is so high that it can even threaten their power or position, they may not give positive feedback to employees and may even show a negative response as EI increases.

## Data Availability Statement

The original contributions presented in the study are included in the article/supplementary material, further inquiries can be directed to the corresponding author.

## Author Contributions

BD contributed to the mechanism, modeling, and conclusion. XP contributed to the other parts of the manuscript. NJ works with translation and revision. All authors contributed equally to the manuscript.

## Conflict of Interest

The authors declare that the research was conducted in the absence of any commercial or financial relationships that could be construed as a potential conflict of interest.

## Publisher’s Note

All claims expressed in this article are solely those of the authors and do not necessarily represent those of their affiliated organizations, or those of the publisher, the editors and the reviewers. Any product that may be evaluated in this article, or claim that may be made by its manufacturer, is not guaranteed or endorsed by the publisher.
